# Rapid pulsed whole genome sequencing for comprehensive acute diagnostics of inborn errors of metabolism

**DOI:** 10.1186/1471-2164-15-1090

**Published:** 2014-12-11

**Authors:** Henrik Stranneheim, Martin Engvall, Karin Naess, Nicole Lesko, Pontus Larsson, Mats Dahlberg, Robin Andeer, Anna Wredenberg, Chris Freyer, Michela Barbaro, Helene Bruhn, Tesfail Emahazion, Måns Magnusson, Rolf Wibom, Rolf H Zetterström, Valtteri Wirta, Ulrika von Döbeln, Anna Wedell

**Affiliations:** Department of Molecular Medicine and Surgery, Science for Life Laboratory, Center for Molecular Medicine, Karolinska Institutet, Stockholm, Sweden; Centre for Inherited Metabolic Diseases, Karolinska University Hospital, Stockholm, Sweden; Department of Laboratory Medicine, Karolinska Institutet, Stockholm, Sweden; Department of Biochemistry and Biophysics, Science for Life Laboratory, Stockholm University, Box 1031, 171 21 Solna, Sweden; School of Biotechnology, Science for Life Laboratory, KTH Royal Institute of Technology, Box 1031, 171 21 Solna, Sweden

**Keywords:** Bioinformatics, Clinical diagnosis, Inborn Errors of Metabolism, Mendelian disease, MPS, WGS

## Abstract

**Background:**

Massively parallel DNA sequencing (MPS) has the potential to revolutionize diagnostics, in particular for monogenic disorders. Inborn errors of metabolism (IEM) constitute a large group of monogenic disorders with highly variable clinical presentation, often with acute, nonspecific initial symptoms. In many cases irreversible damage can be reduced by initiation of specific treatment, provided that a correct molecular diagnosis can be rapidly obtained. MPS thus has the potential to significantly improve both diagnostics and outcome for affected patients in this highly specialized area of medicine.

**Results:**

We have developed a conceptually novel approach for acute MPS, by analysing pulsed whole genome sequence data in real time, using automated analysis combined with data reduction and parallelization. We applied this novel methodology to an in-house developed customized work flow enabling clinical-grade analysis of all IEM with a known genetic basis, represented by a database containing 474 disease genes which is continuously updated. As proof-of-concept, two patients were retrospectively analysed in whom diagnostics had previously been performed by conventional methods. The correct disease-causing mutations were identified and presented to the clinical team after 15 and 18 hours from start of sequencing, respectively. With this information available, correct treatment would have been possible significantly sooner, likely improving outcome.

**Conclusions:**

We have adapted MPS to fit into the dynamic, multidisciplinary work-flow of acute metabolic medicine. As the extent of irreversible damage in patients with IEM often correlates with timing and accuracy of management in early, critical disease stages, our novel methodology is predicted to improve patient outcome. All procedures have been designed such that they can be implemented in any technical setting and to any genetic disease area. The strategy conforms to international guidelines for clinical MPS, as only validated disease genes are investigated and as clinical specialists take responsibility for translation of results. As follow-up in patients without any known IEM, filters can be lifted and the full genome investigated, after genetic counselling and informed consent.

**Electronic supplementary material:**

The online version of this article (doi:10.1186/1471-2164-15-1090) contains supplementary material, which is available to authorized users.

## Background

Massively parallel DNA sequencing (MPS) is revolutionizing diagnostics of inherited diseases, and whole-exome sequencing (targeted capture and sequencing of all coding parts of the genome, WES) has recently entered the clinic [1]. Interrogation of all protein-coding genes results in a high diagnostic yield, but also leads to identification of genetic variants of unclear relationship with the disease under investigation as well as secondary (incidental) findings. Clinical WES therefore requires thorough genetic counseling to prepare subjects for unexpected findings and to explain results. An alternative approach focuses on subsets of monogenic disorders, enabled by filtering of exome data against a panel of selected monogenic disease genes. With a targeted exome approach, the risk for unexpected and secondary findings is greatly reduced and interpretation of results is facilitated. This conforms to guidelines for genetic testing in children [2], which recommend that analysis is limited to the parts of the genome relevant to an individual patient’s presentation, as well as to international recommendations for next-generation sequencing in clinical practice [3, 4].

More recently, whole genome sequencing (WGS) has been used for clinical diagnostics, exploiting the fact that targeted capture prior to sequencing is circumvented, resulting in shorter turnaround times. A strategy was recently described, adopted for neonatal intensive care units (NICUs) [5], enabling 50-hour differential diagnosis of genetic disorders by WGS. This short turnaround time was achieved by technical improvements in sample preparation and sequencing in combination with development of an automated correlation tool for prioritization of clinical information to assist interpretation, and focused on analysis of 591 monogenic diseases with early pediatric presentation.

We report a principally novel approach for clinical WGS based on pulsed analysis. By analyzing pulsed whole genome sequence data in real time, using automated analysis combined with data reduction and parallelization, we show that clinical diagnosis of genetic disorders can be obtained within 15–36 hours. We have applied this rapid pulsed WGS to an in-house developed customized workflow enabling comprehensive diagnosis of all inborn errors of metabolism (IEM) with a known genetic basis, currently represented by 474 genes in our Centre for Inherited Metabolic Diseases (CMMS) Inborn Error of Metabolism gene database (dbCMMS). We have previously validated this diagnostic platform on whole exome data, with an overall diagnostic yield of 60%, using a non-pulsed analysis workflow. For optimal clinical benefit, we have established a collaborative team consisting of experts in pediatrics, neurology, endocrinology, clinical genetics, biochemistry, neuroimaging, dietary treatment and bioinformatics, enabling rapid translation of WGS data directly into individualized clinical treatment even in acute situations. As survival and extent of irreversible brain damage critically depend on time to treatment in acute-onset IEM, this approach has the potential to significantly increase survival and reduce neurological sequelae in affected patients. As a proof-of-concept, two patients who had presented with acute onset neonatal IEM were retrospectively analyzed in a blinded trial with no supplied information on the patient’s phenotypes. The correct disease-causing mutations were identified and presented to the clinical team 15 and 18 hours after start of sequencing, respectively. A third patient who presented during the investigation was also analyzed, with negative findings but allowing assessment of 99% of all exonic bases of the 474 disease genes, making an acute IEM unlikely. In agreement with this finding, extended clinical investigations and analysis of the full exome have been negative and the patient remains without a specific diagnosis.

## Results

The Mutation Identification Pipeline (MIP) [6] together with dbCMMS and the in-house developed software Scout, for visualization of clinical variants, constitute a customized analytical platform designed to enable the adoption of MPS in a clinical setting, meeting health-care requirements and conforming to international guidelines [2–4]. The platform can be adapted to any selection of disease-genes or genomic regions of interest, by substituting dbCMMS with other customized genes or genomic regions. In this study we focused on IEM, a disease area where rapid diagnostics and treatment have dramatic impact on patient outcome, and where we have established an expert center containing all clinical expertise required for rapid translations of findings into individualized treatment. Using this platform we screened all 474 known IEM-causing genes for pathogenic mutations in two patients who had presented with acute disease typical for IEM, in a blinded retrospective study. We also screened one undiagnosed acute patient who presented during the investigation.

### Rapid WGS analysis

In the blinded retrospective analysis of two children with diagnoses that had previously been established by conventional diagnostics, rapid pulsed WGS followed by our pipeline consisting of MIP, dbCMMS, Chanjo and Scout, resulted in presentation of the correct pathogenic variants in both cases. These could be identified, by the investigator even without guidance of the patient’s phenotypes, for all pair-end pulses and ranked highest according to disease causing potential in each clinical candidate variant list. This illustrates the strength of the procedure in rapidly identifying pathogenic mutations, which are presented to the clinical team for evaluation.

The highest scoring variant for Patient 1 was found in the gene *PCCB* encoding for the protein propionyl-CoA carboxylase, beta chain involved in the propionyl-CoA degradation pathway. The nonsynonymous homozygous variant c.1313C > T is very rare, positioned in a conserved region of the genome and the resulting p.Ala458Val amino acid change is predicted to cause loss of protein function. This homozygous variant had been confirmed as diagnostic in the patient by Sanger sequencing at 2 months of age, as the mutation is an established cause of propionic academia [7]. The rapid pulsed WGS test presented the correct pathogenic variant after 15 hours from the start of sequencing preparation in this patient.

An X-linked nonsynonymous variant, c.904C > T, in the *PDHA1* gene was ranked highest in Patient 2. *PDHA1* encodes the Pyruvate dehydrogenase E1 component subunit alpha, which catalyzes the conversion of pyruvate to acetyl-CoA and CO_2_. The variant is described as pathogenic in the common public variant databases. The variant affects four transcripts of *PDHA1* but always gives rise to a p.Arg302Cys amino acid change, predicted to be damaging for protein function. This variant had been found diagnostic in the patient by Sanger sequencing at 25 days of age after extensive biochemical investigations. This had established the diagnosis, as the variant is a known, recurring cause of pyruvate dehydrogenase deficiency [8]. The rapid pulsed WGS test presented the correct disease-causing mutation in this patient after 18 hours from the start of sequencing preparation.

Prospective analysis of Patient 3 did not result in any diagnostic variants in dbCMMS. However, we were able to assess 468 and 640 candidate genes and corresponding Consensus CDS project transcripts [9], respectively, making an acute IEM unlikely.

### Bioinformatic and pulsed analysis performance

**Figure 1:**
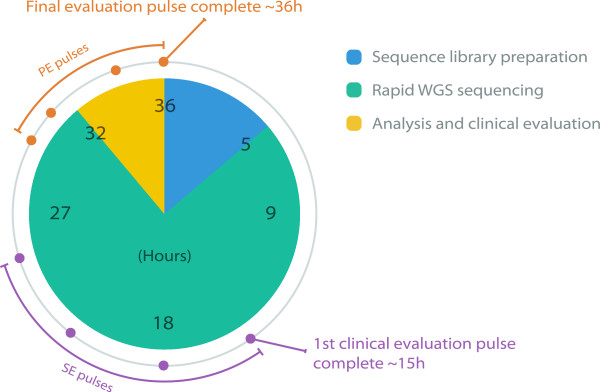
**Pulse distribution and completion.** Timing of pulsed analysis flow from sequencing library preparation to clinical diagnosis. The amount of time spent on each step is only shown for the final evaluation pulse. WGS: whole genome sequencing, SE: single-end, PE: paired-end.

An automated and integrated workflow enabled a first preliminary diagnosis ~15 hours from DNA isolation. The sequential pulsed analysis is able to augment this first preliminary report through reanalysis using: (i) longer reads and (ii) paired-end (PE) reads as the sequencing run progresses. The identified pathogenic mutations were first identified in pulse 1 (single-end (SE) 35 nucleotide (nt) reads) for Patient 1 after approximately 15 hours and in pulse 2 (SE 50 nt reads) for Patient 2 after approximately 18 hours. Subsequent analysis pulses using longer reads and PE data all strengthened the initial findings. All analyzed pulses identified the pathogenic mutations and ranked them at the top of the respective clinical candidate list, except for the initial SE 35 nt reads pulse for Patient 2, which was unable to identify the causal mutation, due to lack of read depth. The final pulse (PE 100 nt reads) was finished within 36 hours of DNA isolation for all patients (Figure 1).

**Figure 2:**
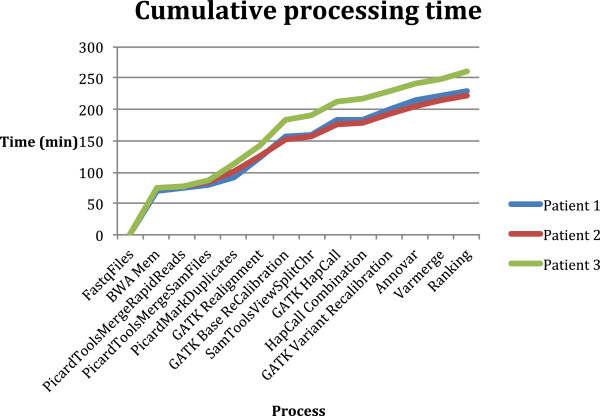
**MIP’s rapid analysis processing time.** Cumulative processing time for the sequence analysis at the final pulse (PE 100 nt reads).

MIP performs the bioinformatics analysis from raw sequences to biologically relevant, annotated and ranked variants for the final pulse in approximately 4 hours (Figure 2; other PE pulses [see Additional file 1: Figure S1]). This rapid data analysis is accomplished by using a parallelization and data reduction scheme aligning all reads to the whole human genome and selecting only data mapping to the exome (or any other user defined region), after the initial alignment step. This novel approach significantly reduces the computational load on downstream processes - substantially speeding up the analysis. The parallelization also ensures that the alignment can be performed efficiently and independently of the depth of sequencing - as the number of nodes scales with the amount of generated sequence data. However, no more than 19 nodes (8 cores per node) in the alignment step of any pulse were used in this study. The variant identification and accuracy are naturally quite poor for the first analysis pulse but improves progressively with each additional pulse. However, 99% of called variants in SE pulse 35 and 50 are reproduced in the final PE pulse, showing the quality of variants in early pulses. The observed transition:transversion ratio and heterozygote:homozygote ratio for the final pulse agree well with the published range of 2.8-3.1 for human exomes and 1.25-1.7 for European ethnicity, respectively [10] [see Additional file 2]. The SNP concordant rate with dbSNP129 is greater than 99.7%, demonstrating the high quality of identified variants. The coverage metrics completeness (width and read depth), and diagnostic yield (number of transcripts fully covered at 10x read depth) for dbCMMS and the exome are shown in [see Additional file 3]. The percentage of bases with adequate read depth (to be evaluated in the clinical test) for dbCMMS exons is 83, 96, and 99 for Patients 1, 2 and 3, respectively.

## Discussion

The last few years have seen a paradigm shift in the investigation of inherited disorders, due to technical advances in DNA sequencing. This has tremendous impact for diagnostics, particularly of single-gene disorders.

Inborn errors of metabolism comprise a large number of monogenic disorders disrupting metabolic pathways. Around 500 different IEM-causing genes are known, causing deficient function of e.g., carbohydrate, amino acid, lipid metabolism or mitochondrial oxidative phosphorylation. Clinical presentation varies widely and ranges from acute, neonatal disease to chronic progressive and intermittent forms sometimes presenting as late as in adult life. Any combination of organs can be affected and patients thus show up across most clinical disciplines. As initial symptoms most often are nonspecific, diagnosis is challenging and screening methods need to be employed on wide indications in order to detect patients at early disease stages. Many IEM are treatable, either by general support including protein and fat restriction to remove potentially toxic substrates, glucose infusion to block catabolism reducing potentially toxic intermediates and to prevent cellular energy deficiency, and administration of a cocktail of vitamin cofactors essential for a range of enzymes; or by specific treatment to remove critical compounds or provide essential substrates once the correct diagnosis is established. There is also an intense development of small molecule drugs and recombinant enzyme therapies in this area. For many conditions, the extent of brain damage and neurological sequelae in affected patients correlate directly with timing and accuracy of management in early, critical disease stages. Serial genetic testing is diagnostic in a minority of IEM cases, since the number of potentially responsible disease genes is large even when initial clinical and biochemical evaluations have homed in on specific metabolic pathways.

For the reasons described above, IEM represents a disease area, which benefits immensely from clinical implementation of MPS. For optimal translation into individualized treatment, a cross-disciplinary infrastructure is needed within this highly specialized area of clinical medicine. The Centre for Inherited Metabolic Diseases (CMMS) at the Karolinska University Hospital has established a cross-disciplinary team of clinical specialists in metabolic medicine, paediatrics, neurology, endocrinology, clinical genetics, biochemistry, and bioinformatics. Intensive care units (ICU), neuroimaging specialists, metabolic clinicians and dieticians are available within the same hospital. CMMS serves more than half of the Swedish population with laboratory investigations and expert advice in cases with suspected IEM. Since 1965, CMMS also performs the nation-wide neonatal screening program (“PKU test”), currently comprising 24 different, treatable conditions. There is thus a long-standing, broad experience in diagnosis and management of IEM.

We have previously used our diagnostic platform comprising MIP, dbCMMS, Scout and Chanjo [11] (in-house developed software for coverage calculations of genomic features) on non-pulsed whole exome data (unpublished). In the initial analyses of previously unsolved cases with a high suspicion of IEM based on clinical and biochemical findings, 8/30 (27%) were immediately diagnostic, establishing the robust performance of the platform (Additional file 1: Table S1). For patients who were negative in this clinical IEM test, we proceeded to investigate the full exome, and identified an additional 10 likely novel disease genes, for which functional validation is ongoing. We have thus validated the platform and established its robustness both for clinical diagnostics and for scientific investigations in this selected patient group, with an overall yield of 60%. Importantly, we clearly separate the clinical diagnostic and the research settings, in order to avoid incidental findings and delays in data interpretation of the clinical test, where a rapid turnaround time is essential. Extended, full exome analysis is only performed after thorough genetic counseling and requires written informed consent. This procedure is in full agreement with ethical guidelines for genetic testing in children [2] and follows international recommendations for next-generation sequencing in clinical practice.

As described in this report, we next applied our diagnostic platform to address acute onset cases by developing rapid pulsed WGS. As proof-of-concept, we retrospectively analyzed two cases that had presented with classic acute, neonatal-onset IEM in a blinded trial without guidance of the patients' phenotypes.

Patient 1 suffered from propionic aciduria (PA), one of a group of organic acidurias. PA is caused by deficiency of the mitochondrial enzyme propionyl-CoA carboxylase (PCC), which is required for metabolism of the four amino acids valine, isoleucine, methionine and threonine and of odd-chain fatty acids. PCC is a multimeric protein composed of α and β subunits encoded by the *PCCA* and *PCCB* genes, respectively. The enzyme is biotin-dependent, and a few patients may respond to biotin treatment. PA usually presents clinically as a severe neonatal onset form with ketoacidotic coma, hyperammonemia and convulsions. There are also late-onset and intermittent forms. Patients should follow a very strict diet with limited protein intake and optimized amino acid preparations. Treatment also includes carnitine and alternated cures of antibiotics to reduce intestinal bacteria which produce propionic acid. Patients are at risk of acute decompensation at times of metabolic stress. Early initiated treatment and close monitoring is essential to avoid death, minimize complications such as psychomotor retardation, cardiomyopathy, and acute pancreatitis.

Diagnosis is conventionally made by chromatography of urinary organic acids and plasma acylcarnitines to detect propionic acid and other derived products. This allows for a rapid biochemical diagnosis, which is possible only in a small number of IEM that result in accumulation and secretion of highly specific compounds. The condition can also be identified by newborn screening, by detection of propionyl carnitine on dried filter-paper bloodspots using tandem mass spectrometry. Results from newborn screening are available around day 7 (median), which is sometimes too late to prevent brain damage or even death in severe forms of PA. Conventionally, the diagnosis is confirmed by mutation analysis of the *PCCA* and *PCCB* genes. As described, rapid pulsed WGS indicated the correct diagnosis after 15 hours in Patient 1.

Patient 2 had primary lactic acidosis, an acute neonatal condition resulting from circulatory failure, septicaemia or indicating a severe underlying metabolic dysfunction. Several IEM can be responsible, including e.g., pyruvate dehydrogenase deficiency (PDHD), pyruvate carboxylase deficiency, defects of gluconeogenesis and a wide range of mitochondrial diseases. All these metabolic disorders are genetically heterogeneous. For example, six subtypes of PDHD have been recognized. The most common PDHD results from mutations in the X-linked *PDHA1* gene, which encodes the E1-alpha subunit of the enzyme complex. Unlike most X-linked enzyme deficiencies, this disease frequently causes severe symptoms in heterozygous females.

The PDH complex is essential by transforming pyruvate generated by glycolysis into acetyl-CoA, which may then be used in the citric acid cycle to generate energy and ultimately enable ATP synthesis by the respiratory chain. The symptoms of PDHD are thus due to bioenergetic deficiency together with lactic acidosis. Manifestations range from fatal, severe, neonatal disease to later-onset neurological disorders and symptoms are aggravated by carbohydrate intake.

Diagnosis is conventionally made by demonstrating abnormal enzyme function in cultured cells, measurements of ATP production in isolated muscle mitochondria using pyruvate as a substrate, and/or genetic analysis. Treatment aims at stimulating the PDH complex by cofactor supplementation with thiamine. A small number of patients with mutations in the *PDHA1* gene are thiamine-responsive. A ketogenic diet is used to provide an alternative source of energy than carbohydrates, particularly for the brain, and dichloroacetate can be used to reduce elevated lactate levels. Prognosis is variable but is generally poor in severe cases, and depends critically on early initiation of treatment. Careful monitoring is essential to minimize the risk of metabolic decompensation.

Glucose infusion is a standard acute treatment in suspected IEM before a specific diagnosis has been established, since successful treatment in most conditions critically depends on interrupting catabolism, reducing intake of protein and fat, and supplying energy. This can result in lactic acidosis and careful monitoring of lactate is required. With access to results from rapid pulsed WGS after 18 hours, specific treatment including a ketogenic diet would have been started several days earlier in Patient 2, likely preventing large parts of the patients' brain damage.

The above two examples illustrate typical situations in acute metabolic medicine. Due to the large number of IEM and their nonspecific initial presentations, acute treatment is nonspecific and aims at preventing further damage while diagnostic workup proceeds to successively direct treatment into the right direction. As serial gene testing only can cover a fraction of all known disease genes, workup includes e.g., measurements of metabolites in body fluids, specific enzyme assays in isolated cells, and/or investigations of respiratory chain function after isolation of mitochondria from a muscle biopsy. It may be weeks before a specific diagnosis is ultimately obtained in successful cases and individualized treatment can be initiated. Importantly, many patients never receive a specific molecular diagnosis even though they suffer from a previously described but rare IEM. The pulsed analysis opens up the possibility of a first clinical assessment of sequence variants in hundreds of relevant genes after only ~15 hours from the start of WGS, although the variant sensitivity and accuracy at this stage is quite poor. In this context, preliminary disclosure is highly acceptable as it directs further investigations into the right direction and aids in the choice of treatment. Results from MPS thus form an integrated part of the clinical workup where multiple parameters are followed by the clinical team, and management is continuously optimized until the situation is stable and data are robust. MPS has thus been adapted so it fits into the dynamic, multidisciplinary work-flow of acute metabolic medicine. As a result, a ketogenic diet could have been initiated several days earlier in Patient 2, likely reducing neurological sequelae substantially. Despite the limitations in quality of the preliminary data, it was still sufficient to identify the causal mutations in pulse 1 for Patient 1 and in pulse 2 for Patient 2. After the first pulse, data successively improve and Sanger sequencing is used to confirm the individual mutations.

Although we were unable to identify the causal mutation for Patient 3 we were able to fully assess 95% of Collaborative consensus coding sequencing project (CCDS) transcripts and in total evaluate 99% of exonic bases (including splice dinucleotides) included in the clinical test (dbCMMS). Hence, these are unlikely to harbor the causal mutation and constitute important negative findings. These results are in agreement with the extended clinical evaluations of this patient, which have failed to provide an explanation for the patients' illness despite months of follow-up. Mining the full exome of this patient has also failed to identify a likely monogenic cause of the patients' illness.

In this study, we have shortened the time to results from a standard WES and WGS in a clinical setting from at least a month to a first preliminary clinical report after 15 hours and a final report after 36 hours. These improvements were made possible by (i) the modification to the data extraction scheme of the widely used Illumina Hiseq 2500 instrument that enabled sequencing of a human genome in less than 30 hours and (ii) pulsed automated analysis and (iii) data selection using a clinically predefined subset of genes, in this study dbCMMS, and (iv) using an in-house developed workflow of automated sequence analysis (MIP), assessment of coverage and diagnostic yield (Chanjo) and visualization and interpretation (Scout).

MIP handles all laborious tasks in the MPS analysis, while the clinical evaluation of the results is performed interactively in Scout, which holds information essential for variant interpretation such as allele frequencies, inheritance patterns, predicted effects on protein function, and links to external databases. This user-friendly visualization tool greatly facilitates clinical translation. The variant sensitivity and accuracy are poor for early pulses, but was sufficient to identify the causal mutation in both retrospective patients examined. The exonic variant quality for the complete run is indistinguishable from a “standard” WGS analysis [see Additional files 2 and 3] but accomplished in only ~4 hours compared to the standard procedure of: alignment, realignment, recalibration; variant identification and recalibration; and variant annotation; which takes approximately 1 week.

Further improvements to the automatic analysis in terms of speed, sensitivity and accuracy are expected as both computer power and the underlying algorithms are continuously improving. We have also not tested omitting some of the recalibration steps used in the analysis for greater speed. It is at present unclear how this would affect the overall false positive and negative rates.

We have found our diagnostic platform for IEM to be efficient and reliable, as it quickly generates decisive, actionable results without large problems with ambiguous or secondary (incidental) findings. Due to this robust performance, the test can be scaled up substantially. The addition of rapid pulsed WGS represents a further dramatic diagnostic improvement. Since initial symptoms of IEM are nonspecific, these tests need to be employed on wide indications, for timely diagnosis of IEM as well as for directing clinical investigations in other directions for patients with other causes behind their illness. However, despite the fact that the overwhelming majority of all disease-causing mutations are located in the protein-coding parts of our genome [12], like all genetic tests they are not 100% sensitive. The continued integration of biochemical and genetic investigations is therefore essential, in order not to miss atypical cases as well as to validate genetic variants of unclear functional significance.

A previous study [5], demonstrated a 50-hour differential diagnosis of genetic disorders by rapid WGS. This method required well-described clinical phenotypes broken down into standardized clinical terms that were automatically matched to selected disease genes, to identify potentially causal mutations. Our novel pulsed analysis reduces turnaround time even further. In addition, our approach can incorporate any selection of disease genes or genomic regions in the analysis and uses standard MPS tools (e.g., BWA, SAMTools and BEDTools) to achieve its speed, making the data reduction and parallelization steps easily adoptable in other MPS pipelines. Furthermore, we advocate working with specialized, multidisciplinary, clinical teams, especially in diagnosis of treatable disorders where irreversible damage results from delayed or erroneous treatment. Automated, computer-based matching of clinical symptoms and disease genes can aid in diagnostics, but in metabolic medicine, where disease presentation is nonspecific and a large number of parameters need to be monitored, and where rapid clinical actions are often required, clinical professionals working across disciplines are needed for optimal translation of the high-throughput genetic data all the way into individualized patient management.

## Conclusions

We have applied our novel rapid, pulsed WGS to an in-house developed customized work flow enabling clinical-grade analysis of all inborn errors of metabolism (IEM) with a known genetic basis, currently represented by 474 genes. Using this approach, MPS is adapted so it fits into the dynamic, multidisciplinary work-flow of acute metabolic medicine, greatly improving diagnostics while conforming to ethical guidelines for genetic testing in children, and to international recommendations for exome and genome sequencing in clinical practice [3, 4]. A cross-disciplinary clinical team has been established, enabling rapid translation of genetic findings directly into individual patient management in this highly specialized field of clinical medicine. Clinical presentations of IEM vary widely and early symptoms are nonspecific. As the extent of brain damage in affected patients often correlates with timing and accuracy of management in early, critical disease stages, our novel methodology is predicted to significantly reduce neurological sequelae and improve the outcome in affected patients. Our approach and the tools developed are designed to also facilitate implementation in other genetic disease areas.

## Methods

### Patients

Two individuals, Patient 1 and Patient 2 presenting with classic, acute-onset neonatal IEM, were retrospectively analyzed. To illustrate the strength of the methodology in objectively identifying likely pathogenic variants, the investigators involved in sequencing and data analyses were not informed of their diagnoses. One acutely ill child presenting during the preparation of this manuscript, Patient 3, was prospectively analyzed. The study was approved by the Regional Ethics Committee at Karolinska Institutet and written informed consent was obtained by the guardians of the patients. Because of the risk of detection of incidental findings not related to the specific medical conditions under investigation, and due to the fact that genome sequences are considered as personal identifiers under the Swedish law, the IRB precluded public dissemination of the raw genomic sequences.

### Whole genome sequencing

Genomic DNA was isolated from the patients using the QIAamp DNA blood midi kit (Qiagen) and prepared for rapid WGS using the TruSeq DNA PCR-Free LT kit (Illumina). Briefly, sample preparation was performed with low sample protocol and fragment size 350 bp, all enzymatic steps and clean up was performed according to manufacturer's instructions.

Clustering and pair-end sequencing (2 × 100 sequencing by synthesis (SBS) cycles) were performed on a HiSeq 2500 (Illumina) in RAPID run mode using on board cluster generation according to manufacturer's instructions.

### MIP

**Figure 3:**
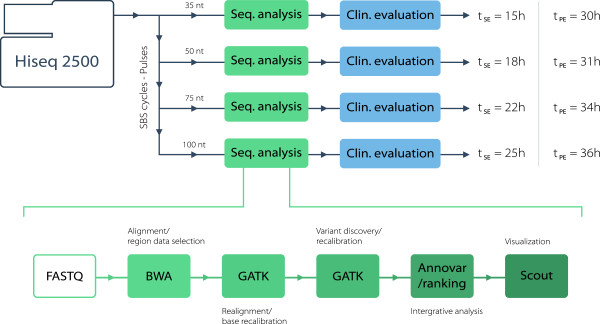
**Sequencing and analysis overview.** Summary of steps used in the pulsed automated bioinformatic analysis, resulting in a final clinical diagnosis within 36 hours. SBS: sequencing by synthesis, Seq: sequencing, Clin: clinical, SE: single-end pulse, PE: paired-end pulse.

The pulsed sequence data were analyzed using the Mutation Identification Pipeline (MIP) v1.3 with the rapid mode setting enabled (Figure 3). MIP is a flexible fully automated sequence analysis pipeline for pedigrees or single samples, which handles WES and WGS data. MIP performs QC of: raw sequences; read mapping; variants, sample integrity (sex and relationship). MIP supports any aligner that inputs fastq files and produces SAM or BAM files. Currently implemented alignment methods are Mosaik and BWA. BAM file pre-processing, variant discovery and filtering are performed with GATK. MIP uses Annovar for standard annotations and also supports any annotation database that can be split by Perl´s split function. Thus, the user has full flexibility to include any annotation on a variant as long as there is a suitable data file available. MIP annotates the genetic inheritance pattern (including compound mutations) and uses a weighted sum model to score and rank variants according to disease causing potential based on a subset of these annotations. Most weight in the rank score model is put on the inheritance pattern, minor allele frequency, gene coding annotation, functional annotation and protein predictions. Thus, MIP retains all variants but prioritizes them according to disease causing potential based on the rank score. MIP allows for selection of variants overlapping genetic panels and analysis of specific genomic regions. MIP [6] is freely and openly available at GitHub.

### Chanjo

Chanjo is an in-house developed analysis tool capable of annotating genomic regions with biological elements e.g. exons, transcripts, gc-content, and calculating sequence coverage across these features. Hence, Chanjo can be used to analyze coverage depth for genomic regions and features, a requirement for clinical-grade analyses, but can also shed light on why some genetic regions are underrepresented by analyzing the associated annotated features. MIP is used in conjunction with Chanjo to generate clinical coverage reports. The coverage reports can be viewed dynamically in a browser or as a static PDF. Chanjo [11] is freely and openly available at GitHub.

### Scout

MIP’s final output i.e., identified, annotated and scored variants, lays the foundation for Scout. Scout organizes and prioritizes the massive information content both from MIP and external resources, providing non-bioinformaticians an easy to use graphical interface accessible via a web browser, and facilitates interpretation and further manual analysis.

### dbCMMS

dbCMMS is a database of genes annotated with clinical information that represent IEM with a known genetic basis, currently represented by 474 genes. dbCMMS has been assembled from our own clinical data gathered from over 25 years of clinical experience, by vetting literature, and public databases. dbCMMS is continuously updated as novel IEM are discovered.

### Sequence analysis

CASAVA v1.8.2 performed demultiplexing and conversion, while the custom script, Pulse_conversion.py [13], extracted raw sequence reads during the sequence run at the predefined SBS cycles: 35, 50, 75 and 100.

Sequence and alignment quality metrics for each patient and PE pulse are available in Additional file 1: Table S2-S3. Variant quality metrics for 2 SE pulses are available in Additional file 1: Table S4. Briefly, the sequence reads were aligned to the whole human genome reference GRCH37 using BWA v0.7.5a [14], while selecting only aligned reads mapping to the exome, as defined by SureSelect Human All Exon V4 (Agilent), for downstream analysis using SAMtools [15], BEDTools [16] and Picard [17]. All coverage metrics were calculated using Chanjo v0.4 [11]. GATK [18, 19] v2.7-2 performed realignment, base recalibration, variant identification, recalibration and genotyping. Annovar [20] v2012-10-23 annotated the variants and MIPs custom scripts ranked them according to pathogenic potential. A summary of annotations and score parameters are available in Additional file 1: Table S5. All variants overlapping dbCMMS v.1.0 were loaded into Scout v1.0 hosted at SciLifeLab for clinical evaluation.

## Electronic supplementary material

Additional file 1: Figure S1: MIP’s rapid analysis processing time. **Table S1.** Results from WES using the MIP-dbCMMS-Scout platform in patients with a high suspicion of IEM. **Table S2.** Sequence and alignment metrics final pulse. **Table S3.** Sequence and alignment metrics PE pulsed SBS cycles. **Table S4.** Variant quality metrics metrics SE pulsed SBS cycles. **Table S5.** Annotations and score parameters used by MIP in the pulsed analysis. (DOCX 83 KB)

Additional file 2: **Variant quality metrics (final pulse; exome variants).** ^1^eval: Evaluated, ^2^Ts: Transitions, ^3^Tv: Transversions, ^4^Het: Heterozygotes, ^5^Hom: Homozygotes, ^6^WGS: Whole genome sequencing, ^7^WES: Whole exome sequencing, ^8^All detected variants, ^9^Detected variants present in dbSNP129. (XLSX 40 KB)

Additional file 3: **Coverage metrics for final pulse.** ^1^Each base covered to at least 10x in CCDS transcripts, ^2^CCDS transcripts fully covered at 10x, ^3^WGS: Whole genome sequencing, ^4^WES: Whole exome sequencing. (XLSX 41 KB)
