# Supplementary material for: Rapid pulsed whole genome sequencing for comprehensive acute diagnostics of inborn errors of metabolism
Source: BMC Genomics. 2014 Dec 11;15(1):1090. doi: 10.1186/1471-2164-15-1090 (PMC4299811; doi:10.1186/1471-2164-15-1090)
Supplement: Supplementary file 1 — Additional file 1: Figure S1: MIP’s rapid analysis processing time. Table S1. Results from WES using the MIP-dbCMMS-Scout platform in patients with a high suspicion of IEM. Table S2. Sequence and alignment metrics final pulse. Table S3. Sequence and alignment metrics PE pulsed SBS cycles. Table S4. Variant quality metrics metrics SE pulsed SBS cycles. Table S5. Annotations and score parameters used by MIP in the pulsed analysis. (DOCX 83 KB) [file 12864_2014_6821_MOESM1_ESM.docx]

**Figure. S1. MIP’s rapid analysis processing time**. Cumulative processing time for 35-75 nt PE pulsed analysis.

## Table S1. Results from WES using the MIP-dbCMMS-Scout platform in patients with a high suspicion of IEM

| **Clinical picture** | **Gene containing pathogenic mutations** |
| --- | --- |
| Mental retardation, dysmyelination, hypermethioninemia, liver dysfunction. | *ADK* |
| Loss of neurological functions from 7 months, rapid deterioration, abnormal signal in white matter. Deceased at 1 year. | *EIF2B* |
| Slowly progressive cognitive impairment and spastic paraparesis, severe white matter atrophy. Debut >40 years. | *EIF2B* |
| Severe neonatal lactacidosis, hypertension, muscular hypotonia, mitochondrial dysfunction (reduced activity complex I). Deceased at 5 months. | *NDUFS1* |
| Liver failure, progressive neurological deterioration, white matter atrophy, mitochondrial dysfunction (reduced activities complex I-IV). | *MPV17* |
| Ataxia, dysartria, epilepsy, mental retardation, mitochondrial dysfunction (reduced activity complex IV). | *ADCK3* |
| Severe encephalopathy, epilepsy, mitochondrial dysfunction (reduced activities complex I,III,IV). | *SERAC1* |
| Late-onset muscle weakness, mild mental retardation, hypothyreosis, renal insufficiency, mitochondrial dysfunction (reduced activity complex I,III,IV). | *PUS1* |

Diagnostic findings were obtained using dbCMMS in 8 of 30 previously unsolved cases (27%). An additional 10 likely novel disease genes were found in the full exome, and are currently being validated (not shown). In all patients with mitochondrial dysfunction, mutations in *mtDNA* and *POLG* had been excluded by Sanger sequencing prior to whole exome sequencing. Respiratory chain function had been evaluated on mitochondria isolated from fresh muscle biopsies.

**Table S2. Sequence and alignment metrics final pulse**

| Sample | Seq^1^ run time (h) | Data size (GB)^2^ | Q30 bases (%) | PF^3^ uniquely aligned reads (%) | Mean target coverage | Target bases 10X (%) | Read duplication (median;%) |
| --- | --- | --- | --- | --- | --- | --- | --- |
| Patient 1 | 27 | 54.9 | 89.5 | 99.8 | 13.8 | 83.0 | 0.10 |
| Patient 2 | 27 | 87 | 94.9 | 99.8 | 27.9 | 99.8 | 0.13 |
| Patient 3 | 27 | 119 | 92.2 | 99.8 | 44.5 | 99.6 | 0.11 |

^1^Seq: Sequence, ^2^GB: Gigabyte, ^3^PF: Passed filter

**Table S3. Sequence and alignment metrics PE pulsed SBS cycles**

| Sample | SBS^1^ cycle (nt) | Seq.^2^ run time  (h) | Data size (GB)^3^ | PF^4^ uniquely aligned reads (%) | Mean target coverage | Target bases 10X (%) | Read duplication (median;%) |
| --- | --- | --- | --- | --- | --- | --- | --- |
| Patient 1 | 75 | 25 | 44 | 99.8 | 6.7 | 15.4 | 0.10 |
| Patient 2 | 75 | 25 | 67 | 99.8 | 13.6 | 83.0 | 0.15 |
| Patient 1 | 50 | 22 | 31 | 99.7 | 5.0 | 4.2 | 0.11 |
| Patient 2 | 50 | 22 | 48 | 99.7 | 5.7 | 8.0 | 0.16 |
| Patient 1 | 35 | 20.5 | 24 | 99.6 | 2.4 | 0.1 | 0.08 |
| Patient 2 | 35 | 20.5 | 36 | 99.6 | 2.7 | 0.1 | 0.10 |

^1^SBS: Sequence by synthesis, ^2^Seq: Sequence, ^3^GB: Gigabyte, ^4^PF: Passed filter

**Table S4. Variant quality metrics metrics SE pulsed SBS cycles**

| Metric | SE 35nt Pulse^1^ | | SE 50nt Pulse^2^ | |
| --- | --- | --- | --- | --- |
|  |  |  |  |  |
| Variant comparison | All^8^ | Known^9^ | All^8^ | Known^9^ |
| No. eval^3^ variants | 2417 | 2273 | 10348 | 9370 |
| dbSNP129 concordant rate (%) | 99.87 | 99.87 | 99.89 | 99.89 |
| No. SNVs | 2415 | 2272 | 10165 | 9265 |
| No. indels | 2 | 1 | 183 | 105 |
| No. SV | 0 | 0 | 0 | 0 |
| Ts^4^:Tv^5^ ratio | 2.93 | 3.05 | 3.09 | 3.14 |
| Het^6^:Hom^7^ ratio | 0.19 | 0.17 | 0.48 | 0.42 |

^1^Patient 1, ^2^Patient 2, ^3^eval: Evaluated, ^4^Ts: Transitions, ^5^Tv: Transversions, ^6^Het: Heterozygotes, ^7^Hom: Homozygotes, ^8^All detected variants, ^9^Detected variants present in dbSNP129

**Table S5. Annotations and score parameters used by MIP in the pulsed analysis**

| Annotation | Rank score parameter | Source |
| --- | --- | --- |
| Ensemble gene ID | No | Ensemble |
| HGNC gene symbol | No | HGNC^1^ |
| HGNC gene name | No | HGNC^1^ |
| HGNC gene name synonyms | No | HGNC^1^ |
| OMIM gene description | No | OMIM^2^ |
| OMIM morbid description | No | OMIM^2^ |
| HGMD accession | Yes | HGMD^3^ |
| HGMD variant type | No | HGMD^3^ |
| HGMD variant associated pubmed ID | No | HGMD^3^ |
| Gene annotation | Yes | ANNOVAR |
| Functional annotation | Yes | ANNOVAR |
| Transcript and protein annotation | No | ANNOVAR |
| Phast cons elements | Yes | ANNOVAR |
| GERP^4^ elements | Yes | ANNOVAR |
| Segmental duplications | Yes | ANNOVAR |
| 1000Genomes MAF^5^ | Yes | ANNOVAR |
| DbSNP MAF^5^ | Yes | ANNOVAR |
| DbSNP nonflagged | Yes | ANNOVAR |
| Esp6500^6^ MAF^5^ | Yes | ANNOVAR |
| SIFT | Yes | ANNOVAR |
| PolyPhen^7^ | Yes | ANNOVAR |
| MutationTaster | Yes | ANNOVAR |
| GERP^4^ | Yes | ANNOVAR |
| LRT^8^ | Yes | ANNOVAR |
| PhyloP^9^ | Yes | ANNOVAR |
| Transfac^10^ | No | ANNOVAR |
| snoRNA & miRNA annotations | No | ANNOVAR |
| GT^11^ call filter | Yes | GATK |
| GT^11^ call | Yes | GATK |
| Genetic inheritance models | Yes | MIP |
| Disease group | No | dbCMMS |
| Clinical db genome build | No | dbCMMS |
| Disease gene model | No | dbCMMS |
| Clinical db gene annotation | No | dbCMMS |

^1^HGNC: HUGO gene nomenclature committee, ^2^OMIM: Online mendelian inheritance in man, ^3^HGMD: Human gene mutation database, ^4^GERP: Genomic Evolutionary Rate Profiling, ^5^MAF: Minor allele frequency, ^6^ESP: NHLBI GO Exome Sequencing Project, ^7^PolyPhen: Polymorphism Phenotyping, ^8^LRT: likelihood ratio test, ^9^PhyloP: phylogenetic p-values, ^10^Transfac: Transcription Factor Binding Sites, ^11^GT: Genotype
